# Psychiatric Deep Brain Stimulation for Refractory Obsessive-Compulsive Disorder: Neurocircuitry, Oscillatory Biomarkers, and Network Modulation

**DOI:** 10.1192/j.eurpsy.2026.11746

**Published:** 2026-07-31

**Authors:** O. Scheuermann, E. Jetter, D. Valle, C. Angelle, B. Carr

**Affiliations:** 1 Department of Psychiatry; 2 College of Medicine, University of Florida, Gainesville, United States

## Abstract

**Introduction:**

Deep Brain Stimulation (DBS) is an advanced neuromodulatory technique that exerts therapeutic effects through direct modulation of brain circuits. In psychiatric disorders, particularly refractory obsessive-compulsive disorder (OCD), DBS is increasingly understood as a network intervention, modulating not just localized regions but large-scale neural dynamics. This poster explores the mechanistic basis of DBS in psychiatry, emphasizing its impact on network coherence, oscillatory synchronization, and electrophysiological biomarkers, all of which can be altered in OCD.

**Objectives:**

To perform a literature review of current DBS studies in neuroimaging, electrophysiology and neural network modulation to evaluate the degree to which DBS for OCD functions as a modulation of large scale network dynamics, rather than a focal or lesion based intervention.

**Methods:**

A literature review was performed, looking at data largely published between 2020-2025, to synthesize current mechanistic insights into psychiatric DBS for OCD. Emphasis was placed on the impact of DBS on neural oscilations and electrophysiological biomarkers, as well as looking at key stimulation targets including the ventral capsule/ventral striatum (VC/VS), nucleus accumbens (NAc), anterior limb of the internal capsule (ALIC), and subthalamic nucleus (STN), all of which provide access to distributed fronto-striatal networks.

**Results:**

DBS primarily targets regions within the cortico-striato-thalamo-cortical (CSTC) loop, a network implicated in affective regulation, cognitive control, and motor planning. It modulates pathological rhythms implicated in OCD, including reductions in theta (4–8 Hz) and beta (13–30 Hz) synchrony linked to cognitive rigidity and compulsivity, while enhancing gamma (30–80 Hz) activity associated with integration and affective regulation. Additionally, DBS restores disrupted phase–amplitude coupling (PAC), supporting hierarchical network coordination. Finally, DBS also recalibrates the temporal coherence of large-scale networks via rhythmic entrainment, cross-frequency coordination, and network reorganization, collectively improving cognitive flexibility and adaptive control.

**Conclusions:**

These findings support the idea that DBS in psychiatry is fundamentally a network intervention, modulating large-scale circuits rather than isolated nodes. Its therapeutic efficacy is mediated by rhythmic modulation, altering oscillatory synchronization, phase coherence, and functional connectivity. As DBS evolves toward closed-loop, biomarker-guided systems, it holds the potential to deliver precision neuromodulation—aligning stimulation with dynamic network state.

**Disclosure of Interest:**

None Declared

